# Amniotic Fluid and Ocean Water: Evolutionary Echoes, Chemical Parallels, and the Infiltration of Micro- and Nanoplastics

**DOI:** 10.3390/toxics13090776

**Published:** 2025-09-13

**Authors:** Antonio Ragusa

**Affiliations:** Obstetrics and Gynecology Unit, Sassuolo Hospital, 41049 Sassuolo, Italy; antonio.ragusa@gmail.com

**Keywords:** abiogenesis, amniotic fluid, microplastics, nanoplastics, One Health, phylogenetic conservation, environmental toxicants

## Abstract

**Background:** Abiogenesis is hypothesized to have occurred in the aquatic environments of the early Earth approximately 3.8–4.0 billion years ago, in oceans containing high concentrations of ions (Na^+^ ≈ 470 mmol/L, Cl^−^ ≈ 545 mmol/L, Mg^2+^ ≈ 51–53 mmol/L, Ca^2+^ ≈ 10 mmol/L, K^+^ ≈ 10 mmol/L, SO_4_^2−^ ≈ 28–54 mmol/L, HCO_3_^−^ ≈ 2.3 mmol/L). Primitive membranes evolved ion-regulatory mechanisms to sustain electrochemical gradients, enabling metabolic activity. **Objectives:** This review compares the composition of amniotic fluid (AF) to seawater, framing AF as a “biological ocean” for the fetus, and evaluates the impact of micro- and nanoplastics (MNPs) on this protected milieu. **Methods:** We synthesized data from published studies on concentrations of and ions and other important substances in AF during pregnancy and compared them with marine values. Reports of MNPs detected in placenta, AF, and human organs were systematically reviewed. **Results:** AF exhibits high ionic similarity to seawater, although the absolute concentrations of ions are lower, reflecting evolutionary conservation. Recent analytical studies identified MNPs in samples of human placenta (4–10 particles per 1 g of tissue), meconium (median 3–5 particles per g), and AF (detectable in >60% of tested samples). Co-exposure to heavy metals, persistent organic pollutants, and endocrine disruptors were reported in 20–40% of maternal–fetal samples. **Conclusions:** The analogy between oceans and AF underscores a conserved evolutionary continuum. However, the infiltration of MNPs into intrauterine environments is a novel toxicological challenge with potential implications for neurodevelopment, immune programming, and epigenetic regulation. Within the One Health framework, protecting AF from anthropogenic contaminants is as critical as safeguarding marine ecosystems.

## 1. Introduction

### 1.1. The Aquatic Origins of Life: The Geochemical and Physiological Context

The Primordial Ocean: Cradle of Life

Pindar 518–438 BC argued that “best is water”; this statement is a good starting point. The leading hypothesis for abiogenesis posits that life first emerged within the aqueous environments of early Earth approximately 3.8–4.0 billion years ago [1,2,3]. These primordial oceans, formed under the influence of intense volcanic activity, hydrothermal fluxes, and atmospheric transformation, were chemically dynamic and devoid of free oxygen [3,4,5]. The result was a mineral-rich, mildly acidic, and reducing aqueous medium capable of supporting complex prebiotic chemistry.

Key electrolytes identified in these early marine environments included sodium (Na^+^), chloride (Cl^−^), potassium (K^+^), calcium (Ca^2+^), magnesium (Mg^2+^), sulfate (SO_4_^2−^), and bicarbonate (HCO_3_^−^). Their relative proportions were governed by geochemical equilibria involving mineral precipitation, hydrothermal interactions, and seawater/rock exchange. Notably, Na^+^ and Cl^−^ were present in significant concentrations, creating a hypotonic milieu favorable for the spontaneous formation of lipid vesicle precursors of protocells [6].

The discovery of mineral-rich hydrothermal vents on the ocean floor has reinforced the plausibility of submarine environments as potential cradles for life. These vents not only provided essential chemical gradients and heat but also catalyzed reactions via naturally occurring transition metal sulfides. Such microenvironments may have supported the polymerization of amino acids, nucleotides, and the formation of protocell membranes, a critical step toward the emergence of cellular life [1].

After the last magma ocean, during the Hadean, the volatile-rich supercritical atmosphere cooled, reacting with the underlying primitive crustal rocks. After the ocean temperature stabilized, a slightly acidic carbonic ocean, rich in Mg and Ca, remained [7]. AF retains a memory of this event, as demonstrated in Table 1.

The recognition that AF is not merely a passive cushion but also an active biochemical reservoir invites a more nuanced understanding of its evolutionary role. Beyond ionic balance, AF contributes to lung maturation through surfactant regulation, gastrointestinal development via fetal swallowing, and immune priming through bioactive molecules such as cytokines and exosomes [8,9].

### 1.2. Evolutionary Continuity: Ionic Parallels in Amniotic Fluid

A remarkable evolutionary echo is observed in the similarity between the ionic composition of early ocean water and that of AF, the protective liquid that envelops the fetus during development [10]. Both media contain comparable concentrations of Na^+^, K^+^, Cl^−^, and HCO_3_^−^ ions essential for osmotic balance, cellular excitability, and pH regulation. This chemical congruence suggests a deep evolutionary conservation of the aqueous environment in which early life, and by extension, human life, develops (Table 1).

The fetal milieu may be viewed as a microcosmic recapitulation of Earth’s ancient seas. The biochemical conditions present in amniotic fluid maintain an optimal ionic and pH environment for fetal cell proliferation, enzymatic activity, and organogenesis. These parallels underscore a physiological continuity that transcends geological eras.

The ionic composition of human amniotic fluid, at approximately 134 mmol/L Na^+^, 110–125 mmol/L Cl^−^, 3–6 mmol/L K^+^, 1.5–2.4 mmol/L Ca^2+^, 1–2 mmol/L Mg^2+^, and 18–23 mmol/L bicarbonate at a pH of 7.0–7.4, closely parallels estimates for the early Archean ocean, which recent models place at a pH of 4.9–6.7, with major cations (Na^+^, K^+^, Ca^2+^, Mg^2+^) in the same order of magnitude as in present-day seawater [7,8,9,10].

This “chemical homology” provides a stable electrochemical gradient essential for enzymatic catalysis, cellular osmoregulation, and the ion-dependent morphogenetic signaling pathways that govern organogenesis in the fetus [9].

From an evolutionary perspective, AF can be regarded as an ontogenetic recapitulation of the prebiotic seas: phylum-conserved mechanisms of ionic homeostasis—mediated by water channels, ion exchangers, and pH buffers—sustain a microenvironment almost indistinguishable in its basic physicochemistry from that which nurtured the earliest marine microbes. By maintaining these primordial conditions, the maternal–fetal unit ensures optimal redox balance, substrate availability, and signal transduction, thereby linking our developmental biology directly to Earth’s Hadean and Archean epochs [11].

## 2. Claude Bernard’s Legacy and the Evolution of Homeostasis

The emergence of homeostatic mechanisms marked a critical step in the evolution of life. Claude Bernard’s concept of the “milieu intérieur”, later expanded by Walter Cannon as “homeostasis”, emphasizes the necessity for internal constancy amid external fluctuation [12,13]. From the earliest single-celled organisms to complex multicellular species, life has depended on the maintenance of ionic gradients and fluid balance.

The semipermeable lipid bilayer of the cell membrane, embedded with transport proteins, permits selective exchange of solutes. Mechanisms such as the Na^+^/K^+^-ATPase pump and various ion channels generate and preserve electrochemical gradients that are indispensable for membrane potential, signal transduction, nutrient uptake, and waste elimination.

Modern extracellular fluids, interstitial fluid, plasma, and cerebrospinal fluid retain an ionic composition reminiscent of ancestral seawater. Enzymatic pathways and cellular functions have evolved under these ionic constraints, further reinforcing the notion that the internal milieu of modern organisms is chemically imprinted by their evolutionary past.

The concept of homeostasis was revolutionary because it introduced the idea that organisms are not passive responders to their environments but rather active regulators of their internal states. This regulation is achieved through complex negative feedback mechanisms involving neural, endocrine, and paracrine signaling systems. For example, the hypothalamic–pituitary–adrenal (HPA) axis plays a central role in modulating physiological responses to stress, while the renin–angiotensin–aldosterone system is essential for maintaining fluid and electrolyte balance [14,15].

In the context of pregnancy, homeostasis acquires an even more critical dimension. The maternal/fetal interface represents a dual system of regulatory interactions, whereby maternal physiological systems adapt to support fetal development without compromising maternal health. Placental endocrine function, fetal fluid exchange, and amniotic fluid composition are all finely tuned through homeostatic mechanisms. Disturbances in these systems, such as in cases of preeclampsia, gestational diabetes, or intrauterine growth restriction, illustrate the consequences of disrupted homeostasis at the maternal/fetal boundary [15].

Moreover, recent advances in systems biology and computational modeling have underscored the multiscale nature of homeostasis, from gene expression and intracellular signaling to organ-level dynamics and whole-body physiology. Homeostatic regulation is now understood to be a product of network-level coordination, often involving redundant pathways that provide resilience against environmental insults. This complexity is particularly evident during fetal development, where precise temporal and spatial regulation of gene networks governs morphogenesis, organogenesis, and immune tolerance.

The thermodynamic and kinetic favorability of prebiotic reactions in hydrothermal vent environments has been increasingly supported by geochemical modeling and experimental simulations [16]. Alkaline hydrothermal systems offer a pH and redox gradient across mineral interfaces that could have served as natural electrochemical reactors, catalyzing the formation of essential organic molecules such as amino acids, nucleotides, and simple peptides. These environments also provided compartmentalization through porous mineral matrices, facilitating the concentration and stabilization of reactive intermediates critical for the origin of life.

Ultimately, Claude Bernard’s insight laid the foundation for modern physiology, biomedicine, and developmental biology. His legacy is particularly salient in the study of maternal/fetal health, where the principles of homeostasis inform our understanding of how intrauterine environments support, or disrupt, the trajectory of human development [12,17]. The emergence of homeostatic control marked a watershed in biological evolution. Claude Bernard’s “milieu intérieur” and Cannon’s “homeostasis” describe active regulation of internal variables—ionic composition, volume, and pressure—despite external perturbations. In a typical mammalian cell, the Na^+^/K^+^-ATPase exports 3 Na^+^ for 2 K^+^ per ATP hydrolyzed, consuming ~30–40% of basal ATP to maintain a resting membrane potential of –70 to –90 mV. Intracellular [Na^+^] (~12 mmol/L) and [K^+^] (~140 mmol/L) contrast with extracellular values of ~145 mmol/L Na^+^ and ~4 mmol/L K^+^, creating the electrochemical gradients essential for nutrient uptake, signal transduction, and volume regulation [12,14,18].

At the systemic level, total body water comprises ~60% of body weight (~42 L in a 70 kg adult), divided between intracellular fluid (~28 L) and extracellular fluid (ECF; ~14 L), of which plasma is ~3 L and interstitial fluid is ~11 L. The volume of cerebrospinal fluid (CSF) is ~150 mL, with a production rate of 0.3–0.4 mL/min (∼500 mL/day) and an ionic composition (Na^+^, 148 mmol/L; K^+^, 2.9 mmol/L; Cl^−^, 120 mmol/L) that again mirrors plasma without the presence of large proteins [19,20].

Negative feedback axes—the hypothalamic–pituitary–adrenal (HPA) and renin–angiotensin–aldosterone (RAAS) axes—operate within narrow quantitative windows: the basal plasma cortisol concentration is usually in the range of 100–200 ng/mL and increases to 500–700 ng/mL under stress; plasma renin activity is typically within 0.5–2.5 ng·mL^−1^·h^−1^, angiotensin II levels are within 30–100 pg/mL, and aldosterone levels are within 100–300 pg/mL, preserving arterial pressure and ECF volume within ±5% of set points [21,22].

In pregnancy, the maternal plasma volume expands by ~40–50% (from ~3 L to ~4.2–4.5 L), and cardiac output increases by 30–50% by mid-gestation. The amniotic fluid volume rises from ~50 mL at 12 weeks to 800–1000 mL at term; fetal swallowing (200–250 mL/day) and urine production (200–500 mL/day) result in a ~100% turnover of AF volume every 3–5 days. Disruption of these homeostatic balances underlies disorders: preeclampsia, gestational diabetes, and intrauterine growth restriction affect ~5%, ~7%, and ~8% of pregnancies, respectively [23,24,25,26,27,28].

Geochemical models of alkaline hydrothermal vents indicate natural proton-motive forces—ΔpH ≈ 1–2 units across Fe–S mineral membranes—equivalent to ΔG ≈ 15–30 kJ/mol, sufficient to drive prebiotic synthesis of amino acids and nucleotides within porous chimneys. Thus, from single cells to complex eukaryotes and human pregnancy, homeostasis reflects an unbroken chain of physicochemical constraints inherited from the Earth’s earliest oceans [29,30].

## 3. Physiological Implications: Water Compartments and Dynamic Equilibrium

Water, the fundamental medium of biochemical reactions and molecular transport, comprises approximately 60% of the human body by weight, with variation depending on age, sex, and body composition. Intracellular fluid accounts for roughly two-thirds of total body water, while extracellular fluid, including interstitial fluid, plasma, and transcellular fluids, constitutes the remaining third.

The dynamic exchange between these compartments is governed by osmotic gradients, hydrostatic pressures, and membrane permeability. Water homeostasis is intricately regulated by neuroendocrine mechanisms involving the hypothalamus, antidiuretic hormone (ADH), renin–angiotensin–aldosterone system (RAAS), and atrial natriuretic peptide (ANP). These pathways maintain plasma osmolality, blood volume, and systemic arterial pressure [31,32,33,34,35,36].

Interstitial fluid serves as a crucial intermediary between blood plasma and the intracellular environment, facilitating nutrient delivery, waste removal, and signal transduction. Its composition closely mirrors that of plasma, except for large proteins that are generally retained within the vasculature. This similarity underscores the fluid’s role as a physiological conduit and its evolutionary continuity with marine environments.

Moreover, the analogy between interstitial fluid dynamics and seawater circulation in porous substrates highlights an evolutionary adaptation: both systems maintain chemical gradients necessary for life by enabling diffusion-driven transport. Even minute disturbances in electrolyte balance, such as those caused by dehydration, fluid overload, or toxicant exposure, can disrupt cellular function, alter membrane potentials, and impair organ systems [17,20].

In the fetal context, amniotic fluid represents a unique extracellular compartment that supports growth and development. It functions not only as a cushion against mechanical trauma but also as a critical regulator of temperature, hydration, and biochemical signaling. The fetal swallowing of amniotic fluid contributes to gastrointestinal tract maturation and renal excretion, which in turn influences amniotic fluid volume. This cyclical exchange exemplifies a tightly regulated aquatic microenvironment shaped by homeostatic principles.

Ultimately, the orchestration of fluid compartments, from the cellular to the systemic level, reflects a complex yet evolutionarily conserved mechanism for sustaining life in a water-based milieu.

The stability of this fluid architecture, like that of early marine habitats, ensures cellular viability and organismal integrity. Even minor perturbations in electrolyte balance can lead to profound physiological consequences, illustrating the fragile equilibrium inherited from our aquatic ancestry [37,38]. Water comprises approximately 60% of total body weight in adults (~42 L in a 70 kg individual), divided into intracellular fluid (ICF; ~28 L, ~40% BW) and extracellular fluid (ECF; ~14 L, ~20% BW). The ECF is further divided into interstitial fluid (~11 L, ~15% BW) and plasma (~3 L, ~5% BW). Plasma osmolality is tightly maintained at 280–295 mOsm/kg by the concerted action of antidiuretic hormone (ADH; plasma levels 1–5 pg/mL), the renin–angiotensin–aldosterone system (RAAS; plasma renin activity 0.5–2.5 ng·mL^−1^·h^−1^), and atrial natriuretic peptide (ANP; 20–77 pg/mL), thereby preserving blood volume and arterial pressure.

Interstitial fluid electrolyte concentrations (Na^+^ ~140 mmol/L; Cl^−^ ~103 mmol/L; K^+^ ~4 mmol/L; Ca^2+^ ~1.2 mmol/L; Mg^2+^ ~0.7 mmol/L) closely mirror those of plasma except for large proteins and maintain osmotic gradients analogous to marine salinity (salinity 35 PSU: Na^+^ ~468 mmol/L; Cl^−^ ~545 mmol/L). This “chemical homology” underpins diffusion-driven nutrient delivery, waste removal, and morphogenetic signaling throughout evolution.

In the fetal context, the amniotic fluid volume increases from ~50 mL at 12 weeks to 800–1000 mL at term; its ionic composition (Na^+^ 135–150 mmol/L; Cl^−^ 100–110 mmol/L; K^+^ 4–6 mmol/L; Ca^2+^ 2.2–2.6 mmol/L; Mg^2+^ 0.9–1.2 mmol/L) and osmolality (280–300 mOsm/kg) maintain a pH of 7.0–7.4, optimizing enzymatic activity, cell proliferation, and organogenesis. Fetal swallowing (~200–250 mL/day at term) and renal excretion (~200–500 mL/day) create a dynamic volume turnover that parallels seawater circulation in porous substrates, thus preserving a self-regulating aquatic microenvironment.

Even minor disturbances—such as dehydration (<1% TBW loss) or fluid overload (>2% TBW gain)—alter plasma osmolality by ±5 mOsm/kg, shifting the cell volume by ~3–5% and impairing membrane potentials (ΔEₘ ≈ 8–15 mV). This sensitivity reflects a deeply conserved dependence on stable aqueous milieus, echoing the conditions of Earth’s primordial oceans [39,40,41,42,43,44,45].

**Table 1:** Comparison of composition of substances in seawater and human amniotic fluid (2nd–3rd trimester).

Substance	Seawater (mmol/L)	Amniotic Fluid (mmol/L, 2nd–3rd Trimester)	Reference
Na^+^	≈470	130–140	[46,47,48,49,50,51,52]
Cl^−^	≈545	100–125	
K^+^	≈10	3–6	
Ca^2+^	≈10	1.5–2.4	
Mg^2+^	≈53	1.0–2.0	
SO_4_^2−^	≈28	0.3–0.5	
HCO_3_^−^	≈2.3	18–23	
δ Minor and trace ions: Br^−^, Sr^2+^, F^−^, H_4_SiO_4_.	Bromide (Br^−^): 0.84 Strontium (Sr^2+^): 0.091 Fluoride (F^−^): 0.068 Dissolved silica (H_4_SiO_4_): 0.02–0.10	Bromide (Br^−^): 0.02–0.08 Strontium (Sr^2+^): <0.01. Fluoride (F^−^): 0.002–0.01 Dissolved silica (H_4_SiO_4_): 0.01–0.03	
pH	7.5–8.5	7.0–7.4	
Salinity (total)	~35‰	~0.5–1.5‰	[10,47,51,52]
Dissolved gases (O_2_, CO_2_, N_2_)	Present (O_2_ often 150–300 μmol/kg)	Present at physiological partial pressures	[47,52,53]
Urea	Present (μM) range; variable by region)	(~4–7 mmol/L) variable in gestation.	[39,47]
Glucose	Trace (nM–μM; rapidly consumed)	≈15–40 mg/dL; <10 mg/dL suggests inflammation	[54,55,56]
Lipids (e.g., lecithin and sphingomyelin)	Present	Traces	[55,56,57,58,59]
Cell	Phytoplankton, prokaryotes, microeukaryotes	Fetal epithelial cells; leukocytes	[10,60,61]
RNA	RNA virus	cfRNA	[59,60,61,62,63,64,65,66,67,68,69,70,71,72,73,74,75,76,77,78]
DNA	eDNA 0.1–88 µg/L up to tens of µg/L; exceptional cases (coastal hotspots) ~5000 µg/L.	Total cfDNA in the range of 50–300 µg/L, with ~10–20% of fetal origin.	
Proteins	0.1%	1–3% total volume	
ϕ Bacteria	10^5^–10^6^ cells/mL (e.g., Prochlorococcus, Pelagibacter)	Traditionally sterile; recent studies report trace DNA, likely contamination? *	
ϕ Viruses	≈10^7^ particles/mL (mostly bacteriophages)	Absent unless there is a maternal–fetal infection (CMV, parvovirus, Zika)	
ϕ Fungi/Other eukaryotes	Marine yeasts, saprophytic fungi, protists	Rare; usually pathological (e.g., Candida in chorioamnionitis)	
Microplastics/Nanoplastics	From ~10^−7^ to 10 particles/L globally (wide range), with well-documented cases ≈2.2 particles/L for 32–651 µm in the Atlantic.	Presence is documented, but available works do not yet report comparable volumetric values; one report indicates ~1.5 particles per sample (volume not reported), and data remain preliminary	[79,80,81,82,83,84]

Note: Seawater values consider standard composition at salinity 35‰. Amniotic fluid values are typical ranges for the 2nd–3rd trimester of an uncomplicated pregnancy. δ In addition to the major electrolytes, seawater and amniotic fluid contain minor and trace ions. In amniotic fluid, available data are sparse and mainly extrapolated from maternal plasma. These values indicate that although the ionic spectrum is broadly comparable, the concentrations of minor ions in amniotic fluid are markedly lower and less well characterized than those in seawater. cfRNA: cell-free fetal RNA; eDNA: extracellular DNA; cfDNA: cell-free fetal DNA. * Sterility paradigm debated [68,69,70,71,72,73,74,75,76,77,78,79,80,81,82,83,84,85,86,87]. ϕ In oceans, microorganisms are vital for ecological cycles [63,64,65].

Table 1 indicates that the ocean represents an ecosystem colonized by microorganisms. These include bacteria, such as *Prochlorococcus* and *Pelagibacter*, which are known to be very abundant organisms and participate in biogeochemical cycles. Marine viruses, especially bacteriophages, play a role in regulating microbial dynamics and nutrient recycling. It is estimated that there are about 10^7^ viral particles per milliliter of seawater. Fungi and microeukaryotes, including marine yeasts and saprophytic fungi, are involved in the degradation processes of organic matter. These microorganisms are part of the marine food web and maintain an ecological balance. Seawater contains both free and intracellular genetic material.

Estimates indicate the presence of bacteria and archaea equal to about 10^5^–10^6^ cells/mL, viruses around 10^7^ particles/mL and dissolved extracellular DNA (eDNA) with typical values of about 0.5–5 μg/L, corresponding to about 0.00005–0.0005% of the total mass of water. Relative to the total content, DNA represents less than 0.001%, but on a global scale, it is equivalent to hundreds of millions of tons of DNA present in the oceans. From an ecological point of view, this DNA contributes to the marine genetic heritage and horizontal transformation.

In physiological pregnancy, amniotic fluid is traditionally considered sterile, although metagenomic studies, conducted since 2014, report the presence of microbial DNA at very low concentrations; however, there is debate, and some authors attribute these results to possible laboratory contamination. The presence of cell-free fetal DNA (cffDNA) is documented: it originates from trophoblastic apoptosis and constitutes a small fraction of the total extracellular DNA. In percentage terms, cffDNA can represent 10–20% of extracellular DNA in amniotic fluid, while in maternal plasma it is generally found between 3 and 10%. The overall concentration of DNA in the amniotic fluid is variable, but usually in the order of ng/mL, making it much lower than in the ocean in terms of microbial biomass. In the case of intrauterine infection (such as chorioamnionitis or viral infections), the amount of microbial DNA grows to detectable levels, while always remaining lower than the human component, both maternal and fetal.

### 3.1. In Summary

Ocean: Abundant total DNA (cellular + extracellular), especially of microbial and viral origin; eDNA is fundamental in the ecosystem.

Amniotic fluid: Predominantly human DNA (maternal and fetal); microbial DNA is very rare (<0.01%) and often discussed.

Orders of magnitude: Microbial DNA is much more abundant in the ocean than amniotic fluid; in the latter, fetal DNA is most important for clinical uses. Amniotic fluid has more DNA per liter than the oligotrophic open sea, but less than some coastal areas rich in life.

### 3.2. Environmental Toxicants and MNPs: A New Threat to Life’s Aquatic Niche

Prenatal exposure to environmental toxicants has long been recognized as a major determinant of adverse maternal and fetal outcomes [88]. Compounds such as heavy metals (e.g., lead and mercury), persistent organic pollutants (e.g., dioxins and PCBs), nicotine, and pharmaceutical residues can induce oxidative stress, disrupt endocrine signaling, and alter epigenetic regulation during key windows of fetal vulnerability [88].

More recently, microplastics (MPs), plastic particles between 0.1 µm and 5 mm in size, and nanoplastics (NPs), synthetic polymer particles ranging from 1 to 1000 nm in size [89,90,91,92,93,94], have emerged as novel and pervasive environmental contaminants. Originating from both the fragmentation of larger plastic debris and primary microplastic products, these particles have been detected in marine, freshwater, terrestrial, and atmospheric compartments [95]. Their ubiquitous presence in the biosphere increases the likelihood of human exposure through ingestion, inhalation, and dermal contact [96] (Table 2, Table 3 and Table 4).

Every year, an estimated 11 million metric tons of plastic waste enter the world’s oceans, a figure projected to nearly triple by 2040 without urgent mitigation efforts [128].

Plastic is responsible for significant damage to human health, the economy, and the environment. This damage occurs at every stage of its life cycle, from the extraction of coal, oil, and gas (which are the main raw materials in 98% of plastic materials) to the recycling process and to its final disposal. The pervasiveness of plastic in all environments is well documented [129].

The greatest vulnerability to the toxic effects of pollutants occurs during fetal life and in the first years of life. In this period, with differentiated times, maturation of the following occurs: (1) organs and systems; (2) metabolic, endocrine, and immunological systems; (3) hepatic and renal detoxification mechanisms; (4) the skin and the blood–brain barrier [130]. Once internalized, MNPs can cross epithelial barriers, enter systemic circulation, and accumulate in organs, including reproductive tissues. Importantly, they also act as vectors for co-contaminants, adsorbing hydrophobic chemicals such as phthalates, polycyclic aromatic hydrocarbons, and heavy metals onto their surfaces. These adsorbed toxicants may be co-delivered into sensitive biological compartments, amplifying their harmful potential [131,132].

Recent studies have confirmed the presence of MNPs in critical maternal and neonatal matrices, including placenta [133], amniotic fluid [134], and human breast milk [135].

These findings challenge the assumption that the intrauterine and early postnatal environments are insulated from environmental pollution. The concept of the fetus developing in a pristine sanctuary is increasingly untenable in the face of accumulating evidence that synthetic particles permeate the maternal/fetal interface.

The potential for MNPs to interfere with fetal programming, immune system maturation, long-term metabolic outcomes, and the vitality of trophoblastic cells [136] is of growing concern [137]. An association was found between the presence of microplastics in meconium and reduced microbiota diversity [137]. Other studies showed that microplastic levels in the placenta correlated with reduced birth weight, Apgar scores at 1 min, and reduced fetal growth in IUGR pregnancies and neurologic problems [138,139]. Furthermore, the presence of MPs in the placenta was correlated with premature birth [79].

Emerging data suggest that prenatal and perinatal exposure to MNPs may have profound effects on neurodevelopment [140,141]. The developing fetal brain is highly vulnerable to environmental insults due to the ongoing processes of cell proliferation, migration, differentiation, synaptogenesis, and myelination. MNPs, along with the chemical contaminants they carry, have been shown in animal models to cross the blood–brain barrier and accumulate in brain tissues, where they induce oxidative stress and neuroinflammation [140,141,142,143,144]. This raises the possibility that MNP exposure could interfere with the molecular signaling pathways essential for neurodevelopment, including those mediated by neurotrophic factors, neurotransmitters, and endocrine signals [145].

Animal studies report changes in behavior, learning capacity, and synaptic plasticity in offspring exposed to MNPs in utero, supporting the hypothesis that these particles may act as neurodevelopmental disruptors [141,142,143,144].

In this context, the amniotic fluid, so chemically like ancient seawater, has become a repository for anthropogenic contaminants, reflecting not only our evolutionary past but also our modern ecological impact.

Longitudinal human studies are urgently needed to confirm these associations and to elucidate the dose–response relationship between MNP burden and health outcomes across the lifespan (Table 5).

Environmental factors significantly influence the fragmentation, bioavailability, and toxicity of MNPs in both marine and terrestrial ecosystems. These factors, including temperature, UV radiation, pH, oxygen levels, and the presence of other pollutants, can alter the physical and chemical properties of MNPs, affecting their ability to be ingested by organisms and interact with biological systems and, ultimately, their toxic effects [153,154].

In marine ecosystems, wave action and sunlight can break plastic waste down into smaller particles that are ingested by fish and other marine life, potentially leading to the bioaccumulation and biomagnification of toxins. In terrestrial ecosystems, agricultural practices can introduce MNPs into soil, impacting soil structure, plant growth, and the health of soil microorganisms. Runoff from land can also carry MNPs into freshwater ecosystems and ultimately to the oceans.

In summary, a complex interplay of environmental factors influences the fate and effects of MNPs in both marine and terrestrial ecosystems, highlighting the need for a comprehensive understanding of these interactions to mitigate the impacts of plastic pollution.

## 4. The One Health Paradigm: Linking Ocean and Amniotic Fluid

The One Health paradigm offers a unifying conceptual framework that recognizes the interdependence of human, animal, and environmental health. Originally applied to zoonotic disease surveillance and ecosystem preservation, the One Health approach has expanded to include the study of environmental pollutants and their systemic effects across biological domains [155,156].

**Figure 1:**
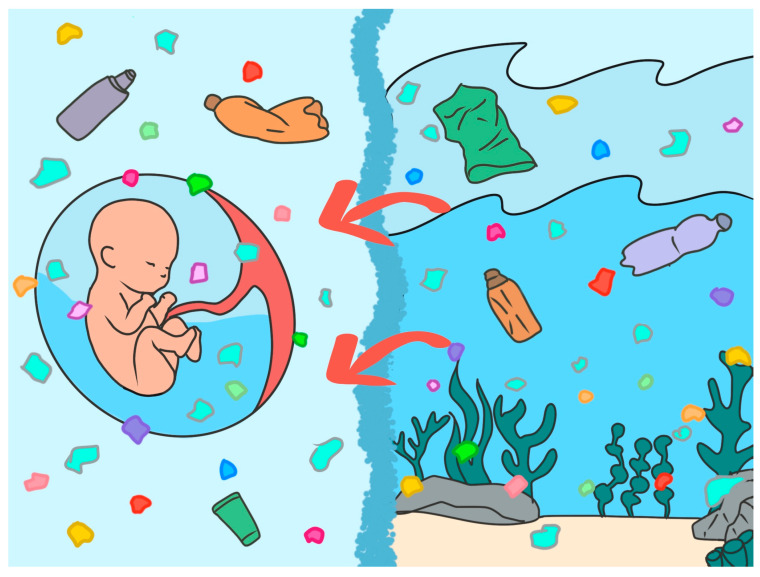
MNPs invasion of vital fluids: seawater and amniotic fluid.

MNPs exemplify the need for this integrative perspective. Their widespread environmental dissemination and biological accumulation demonstrate that synthetic particles do not respect taxonomic, geographical, or physiological boundaries. What is found in the depths of the ocean is now also detected within the intrauterine environment (Figure 1).

One Health is not merely a conceptual tool but a practical model that enables the detection, monitoring, and mitigation of complex contaminant pathways across marine, terrestrial, and clinical environments. The trophic transfer of MNPs across marine food webs, from plankton to fish to humans, illustrates the continuity of exposure across species and ecosystems [157,158]. Likewise, the presence of the same contaminants in umbilical cord blood, amniotic fluid, and placenta confirms the intergenerational and cross-species implications of pollution [135,136,137].

This model calls for transdisciplinary collaboration, integrating marine biologists, obstetricians, epidemiologists, chemists, and environmental engineers. Current research silos often fail to capture the continuity between oceanic plastic load and fetal plastic exposure. A unified surveillance system, anchored in the One Health framework, could map this continuum, enabling early warning systems and regulatory responses [159,160,161]. Incorporating One Health into educational curricula, from secondary school to medical training, can cultivate a new generation of professionals who understand that fetal well-being is linked to environmental stewardship. The paradigm also has profound ethical implications: it challenges anthropocentric notions of health and invites a planetary ethic of responsibility, acknowledging that protecting the fetus also means protecting the planet that nourishes the fetus itself [162].

The amniotic fluid and ocean water are chemically and symbolically linked: both are aqueous matrices that sustain life, buffered by evolutionary processes and now disrupted by human activity. The translocation of plastic particles from marine systems into fetal compartments epitomizes the global reach of pollution and its implications for intergenerational health [163,164,165].

Addressing this crisis demands coordinated, cross-sectoral efforts involving marine biologists, obstetricians, toxicologists, public health officials, and policymakers. By adopting a One Health strategy, we can better understand and mitigate the continuum of exposure that bridges ecosystems and embryonic development [166,167].

## 5. Conclusions: Protecting the Fluids of Life

Amniotic fluid is not merely a by-product of pregnancy; it is an evolutionary innovation that reflects the primordial marine environments from which life originated. Its ionic composition, buffering capacity, and biologically active constituents create an ideal microenvironment for fetal development, an echo of Earth’s early oceans.

Today, both vital fluids, ocean water and amniotic fluid, are contaminated by synthetic pollutants, particularly MNPs. This dual pollution of planetary and intrauterine waters serves as a stark reminder of the interwoven fates of ecology and human health.

Safeguarding future generations requires an urgent commitment to reducing plastic pollution, enhancing monitoring systems for emerging contaminants, and protecting the aquatic environments that cradle life in its earliest stages. In doing so, we preserve not only the health of individuals but also the integrity of the evolutionary lineage that connects all life forms.

In addition, the degradation of these life-sustaining fluids represents not only a medical and environmental emergency but also a bioethical challenge. If intrauterine life is now vulnerable to artificial contaminants previously confined to industrial waste, then the scope of perinatal care must broaden to include environmental stewardship.

**Figure 2:**
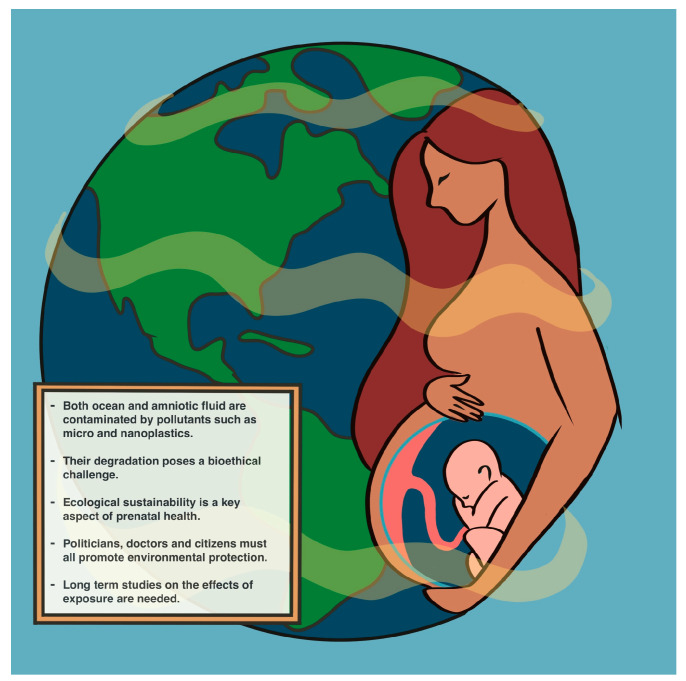
Protecting the fluids of life.

Medical professionals, especially obstetricians and neonatologists, must now advocate for ecological sustainability as a dimension of prenatal health (Figure 2). Hospitals and labs cannot be isolated from the ecosystem; rather, the integrity of pregnancy outcomes is inextricably linked to the integrity of the biosphere.

Furthermore, transdisciplinary collaboration must evolve from academic rhetoric to policy enforcement. The acknowledgment of MNP exposure as a public health threat requires integration of clinical data, toxicological thresholds, and regulatory frameworks.

Long-term cohort studies tracking prenatal exposure to plastic derivatives and associated outcomes in neurodevelopment, immune regulation, and metabolic programming must become standard scientific practice [168,169,170].

Above all, the amniotic fluid must not become the final destination for humanity’s waste. Its contamination is not merely symbolic; it is mechanistically implicated in disruptions to fetal physiology and developmental trajectories.

Very recently, Cordiner et al. used the Atacama Large Millimeter/submillimeter Array (ALMA) to map heavy water (HDO) in the Halley-type comet 12P/Pons–Brooks ‘Devil Comet’. Their analysis showed that the deuterium-to-hydrogen (D/H) ratio of cometary water is consistent with that of the Earth’s oceans. The maps are consistent with outgassing of both H2O and HDO directly from the nucleus, and they imply a coma D/H ratio (for water) of (1.71 ± 0.44) × 10^−4^. This is at the lower end of the range of values previously observed in comets and is consistent with D/H in the Earth’s ocean water. This means that Halley-type comets could have contributed significantly to Earth’s water inventory. In addition, this study challenges the prevailing view that carbonaceous asteroids were the dominant source of terrestrial water. With ALMA’s ability to measure isotopic compositions in comets remotely with high precision, their study contains strongest evidence that comets delivered water to Earth, given that the water found on Comet 12P/Pons-Brooks is “virtually indistinguishable” from water found on Earth.

This study advances our understanding of a universal common origin, as water is the fundamental matrix of life. If the water composing our world is derived from space, it reinforces the notion that all living systems are ultimately formed from stardust [171]. Although the argument on the origins of water remains open, the fact remains that to defend the fluids of life is to defend the origin, continuity, and future of life itself. From the geological depths of hydrothermal vents to the intimacy of the womb, water has been the universal medium of existence, and we must now protect it with equal universality and urgency [172,173].

To echo Pindar’s words, ‘best is water,’ the parallel between marine and intrauterine environments reveals that water is not only the origin of life but also its enduring safeguard.

## 6. Future Directions and Implications for Policy and Research

The growing detection of MNPs in critical biological fluids, including amniotic fluid, raises urgent concerns for fetal development, reproductive health, and long-term disease trajectories. This emerging evidence mandates coordinated action on several levels:

Clinical Research: There is a pressing need for prospective cohort studies and toxicological models to investigate the effects of MNPs and associated endocrine disruptors during pregnancy. Particular attention should be given to fetal neurodevelopment, immune programming, and epigenetic modulation. Such studies should utilize integrative approaches combining metabolomics, transcriptomics, and epigenomics to characterize fetal responses to plastic-derived contaminants. Investment in green chemistry is essential for developing truly biodegradable, non-toxic alternatives to current polymer-based plastics. Research should prioritize the design of materials that degrade into inert, non-bioaccumulative products [174].

Analytical Methods: Standardized, sensitive, and reproducible methods must be developed to detect and quantify MNPs in human biological matrices, including amniotic fluid, placenta, and cord blood. New advancements in high-resolution spectroscopy, e.g., pyrolysis-GC/MS, micro-FTIR (gas chromatography–mass spectrometry with pyrolysis; *micro* Fourier-transform infrared), and nanoscale imaging should be incorporated into standardized protocols to detect ultrafine plastic particles in biological matrices. These technologies will improve detection sensitivity and specificity, reducing false negatives in fetal and neonatal samples. Regulatory bodies such as the FDA, EMA, and WHO must define acceptable thresholds for MNP concentrations in human biological matrices. This will require interdisciplinary consensus on toxicity benchmarks and risk assessment methodologies, facilitating global harmonization of standards. Establishing open-access databases for MNP concentrations in environmental and clinical samples would enhance transparency and global cooperation. These repositories could include metadata on sampling techniques, population demographics, and geographical distribution, fostering meta-analyses and public health modeling.

Environmental Policy: Governments and regulatory agencies must urgently implement stricter controls on plastic production and disposal, with an emphasis on banning non-essential single-use plastics and improving microplastic filtration in wastewater systems. A tax on plastic manufacturing and subsidies for biodegradable alternatives could shift market behavior. Legislative frameworks such as the EU’s REACH (Registration, Evaluation, Authorization, and Restriction of Chemicals) should be adapted globally to include MNPs as emerging contaminants. The recent failure of the UN plastic Geneva negotiations [175] demonstrates that these actions are all absolutely urgent.

One Health Integration: Policy frameworks should embed the One Health perspective, recognizing that protecting marine ecosystems is intrinsically linked to safeguarding intrauterine environments and, ultimately, human reproductive health. Establishing international research consortia linking oceanographers, obstetricians, toxicologists, and environmental chemists is critical for implementing a One Health surveillance system. This system should monitor sentinel species (e.g., plankton, mollusks, marine mammals) and human pregnancy biomarkers in tandem.

Public Awareness: Educational campaigns should inform people about the routes of plastic exposure, its reproductive risks and actionable steps to reduce contact. Integrating MNP-related content into medical and environmental science curricula could prepare the next generation of professionals to address this issue proactively [176].

Understanding the molecular and systemic effects of MNPs during pregnancy will be essential not only for fetal safety but also for redefining our interaction with the synthetic materials that saturate the biosphere.

As Rachel Carson reminded us, “*in nature nothing exists alone”*: the continuity between ocean and amniotic fluid underscores that human health, planetary health, and the origins of life are inseparably intertwined.

## Figures and Tables

**Table 2:** Occurrence, concentration, and analytical methods of detecting MNPs in biological and food matrices worldwide.

Sample Matrix	Location	Contaminant	Concentration (Unit)	Analytical Method	Reference
Mytilus edulis (blue mussel) and Bivalves, Mytilus edulis and Crassostrea gigas	North Sea (cultured) Supermarkets in Brittany, France.	Microplastics	0.36 ± 0.07 particles/g ww. And M. edulis contains on average 0.36 ± 0.07 particles g(−1) (wet weight), while a plastic load of 0.47 ± 0.16 particles g(−1) ww was detected in *C. gigas*.	Micro-FTIR imaging and Raman spectrometer was operated at a laser wavelength of 785 nm (diode), and high-resolution spectra were recorded in three spectral windows. The microscope has 5, 20, and 50 objectives, with spot sizes of approximately 50, 10, and 4 mm, respectively.	[92,93]
Scapharca subcrenata (ark clam)	China (fish market)	Microplastics	10.5 particles/g ww	Micro-FTIR spectroscopy	[93]
Table salt (sea salt)	China (supermarkets)	Microplastics	550–681 particles/kg	FTIR spectroscopy	[94]
Bottled water	Global	Microplastics	325 particles/L	µ-FTIR imaging	[95]
Bottled water	Global	Nanoplastics	240,000 particles/L	SRS microscopy	[95]
Honey	Italy (various origins)	Microplastics	62 particles/kg	Micro-FTIR imaging	[96]
Oreochromis niloticus (Nile tilapia, fillet)	Laboratory RAS	Microplastics	0.14 ± 0.32 µg/g ww	Pyrolysis-GC–MS	[97]
Litopenaeus vannamei (white-leg shrimp)	Thailand (pond)	Microplastics	1.69 ± 0.13 particles/g ww	Micro-FTIR imaging	[98]
Beers (24 brands)	Germany (supermarkets)	Microplastics	2–79 fibers/L	Optical microscopy	[99]
Tea bags in the tea infusions (plastic teabag)	Laboratory simulation	Microplastics	microplastics released from tea bags in the tea infusions ranged from 80 to 1288 pieces (micron-sized) and 0 to 63.755 μg (submicron-sized) per filter bag.	Laser confocal micro-Raman and direct classical least squares and pyrolysis-gas chromatography/mass spectrometry	[100]
White wine (PE stopper)	Europe (retail)	Microplastics	up to 5857 particles/L	Micro-Raman spectroscopy	[101]
Sugar	Various origins	Microplastics	217 ± 123 fibers/kg; 32 ± 7 fragments/kg	Optical microscopy	[102]
Cow’s milk	Italy (supermarket)	Microplastics	204–1004 particles/100 mL	Raman spectroscopy	[103]
Brewed coffee (plastic drip bag)	Laboratory simulation	Microplastics	>10,000 particles/cup	Stereomicroscopy and FTIR imaging	[104]
Soft drinks	Various markets	Microplastics	9 particles/L	FTIR stereoscopy and stereomicroscopy	[105]
Fresh cheese	Italy (retail)	Microplastics	1280 particles/kg	FTIR-ATR imaging	[103]
Ripened cheese	Italy (retail)	Microplastics	1857 particles/kg	FTIR-ATR imaging	

Notes: ww = wet weight; RAS = recirculating aquaculture system; FTIR = Fourier-transform infrared spectroscopy; µ-FTIR = micro-FTIR; ATR = attenuated total reflectance; SRS = stimulated Raman scattering. The pyrolysis-GC–MS result is reported as a mass concentration (µg polymer/g ww), not a particle count.

**Table 3:** Principal sample matrices for inhalation exposure to MNPs, showing sampling environments, typical concentration ranges (or mass fractions), analytical detection methods, and key references.

Sample Matrix	Location	Contaminant	Concentration	Analytical Method	Reference
Indoor residential air	Apartments	Microplastics	Variable	FPA-µFTIR imaging	[106]
Indoor apartments and office air	Apartments	Microplastics	Variable	Gravimetric analysis, stereomicroscopy, and Raman spectroscopy	[107]
Outdoor urban air	Residential streets (China)	Microplastics	2.66 ± 1.76 particles m^−3^	µFTIR imaging	[108]
Public bus cabin air	City buses (multiple cities)	Microplastics	17.3 ± 2.4 particles m^−3^	FPA-µFTIR imaging	[109]
Subway platform air	Metro stations (multiple cities)	Microplastics	5.8 ± 1.9 particles m^−3^	FPA-µFTIR imaging	[110]
PM_2_._5_ fraction of urban air	Outdoor urban monitoring sites	Microplastics (fraction)	3–7% of PM_2_._5_ mass	Review of ambient-PM studies	[111]
Indoor household dust	Households (Australia)	Microplastics	2046 ± 830 particles/g dust	µFTIR imaging	[112]
Plastic recycling plant air	Recycling facility	Microplastics	5.97 106–1.12 × 108 MP m^−3^	fluorescence microscopy analysis	[113]
Waste sorting facility air	Municipal solid waste	Microplastics	From 1.7 to 24.7 N/m^3^ with an average (±standard deviation) of 6.54 ± 5.08 N/m^3^.	Raman spectroscopy	[114]
Textile manufacturing	Czech Republic.	Microfibers	Amount of microplastic fibers released from the fleece fabric increased continuously until the third to fifth washing cycle, after which the released amount was nearly constant.	washing process	[115]
Incineration	12 mass burn incinerators. China	Microplastics	1.9–565 n/kg	Micro-Fourier transform infrared spectroscopy.	[116]
Rural ambient air and forest	Thulamela Local Municipality	Microplastics	Ranging from 90.51 ± 15.19–355.64 ± 47.65 particles/m^2^/day, with an overall average of 211.87 ± 31.44 particles/m^2^/day.	FTIR	[117]

Notes: FTIR = Fourier-transform infrared spectroscopy; µ-FTIR = micro-FTIR; XRF = X-ray Fluorescence; SEM/EDX = Scanning Electron Microscopy/Energy-Dispersive X-ray Spectroscopy.

**Table 4:** Main sample matrices for dermal exposure to MNPs, with sampling contexts, typical concentrations, contaminants, analytical methods and bibliographic references.

Sample Matrix	Location/Context	Contaminant	Concentration	Analytical Method	Reference
Household dust	Private homes	Microplastics	38–120,000 µg/g (median: 5900 µg/g)	Various	[118]
Facial exfoliating scrub (commercial)	UAE market (2019–2020)	Microplastic beads	Up to 6298 ± 1543 beads per g product	FTIR imaging	[119]
Rinse-off cosmetics (face wash, body scrub)	Global survey of PCCPs	Microplastics	Geometric mean 2 162 particles/g; 0.04 g plastic per g product	µFTIR imaging	[120]
Surgical masks (used)	Consumer use	Microplastics/fibers	18.27 ± 4.1 items released per mask	ATR-FTIR spectroscopy	[121]
Beach sand	Coastal sand	Microplastics	Average of 590 ± 360, with 950 ± 100 in the lower wrack zone, 540 ± 40 in the upper wrack zone, and 270 ± 30 in areas between	µ-FTIR.	[122]
Nonwoven wet wipes (mechanical abrasion)	Lab simulation	Microfibers	60–4000 microfibers released per cm^2^	SEM + µFTIR imaging	[123]
Synthetic polyester T-shirt	Machine washes	Microfibers	700,000 fibres could be released from an average 6 kg wash load of acrylic fabric	Abrasion chamber + microscopy	[124]
Agricultural plastic (farmers’ health)	Field (vegetable farm, Italy)	Microplastics		Various	[125]
Playground rubber crumb (sports fields)	Outdoor playgrounds	Micro-/nanoplastics	Up to 30,426 ng/m^3^	SEC-HRMS	[126]
Interior wall paint surfaces	Residential walls	Microplastics, nanoplastics, inorganic nanoparticles		SEM + Raman spectroscopy	[127]

Notes: FPA-µFTIR imaging = Flat Panel Array–micro Fourier Transform Infrared imaging; FTIR = Fourier-transform infrared spectroscopy; ATR = attenuated total reflectance; SEM = Scanning Electron Microscopy.

**Table 5:** Summary of adverse effects induced by MNP exposure in mammalian models and human tissues, indicating plastic type/size, exposure regimen, key outcomes, and analytical methods.

System/Endpoint	Plastic Type and Size	Exposure Regimen	Key Outcomes	Analytical Methods	Reference
Neurological	Polystyrene NPs (~50–100 nm)	Oral gavage in mice, 28 days	Impaired learning and memory; ↑ ROS; lipid peroxidation in hippocampus	Morris water maze, ROS assay, histology	[144]
Endocrine	Polystyrene MPs (5 μm) + Lead	Oral exposure, mice, 35 days	Thyroid hormone disruption (↓ T4, ↑ TSH), altered ovarian steroidogenesis	ELISA, histopathology	[145]
Hepatic	Polystyrene NPs (50 nm)	Oral gavage in mice, 28 days	Hepatic steatosis, ↑ ALT/AST, oxidative stress	Biochemical assays, liver histology	[145]
Renal	Polystyrene MPs (5 μm)	Oral exposure in mice, 8 weeks	Tubular damage, oxidative stress, mitochondrial dysfunction	Histology, oxidative stress biomarkers	[146]
Immunological	Polystyrene MPs (0.5–5 μm)	Oral gavage, mice, 6 weeks	Splenic inflammation, cytokine imbalance (↑ TNF-α, IL-6)	ELISA, flow cytometry	[147]
Cardiovascular	Polystyrene NPs (80 nm)	Intravenous injection, mice, acute	Endothelial dysfunction, ↑ inflammatory markers	Echocardiography, histology	[148]
Reproductive	Polystyrene NPs (50 nm)	Oral exposure, male mice, 35 days	↓ Sperm motility, abnormal morphology, testosterone reduction	Sperm analysis, ELISA, histology	[149,150]
Developmental (Fetal/placental)	Polystyrene NPs (20–200 nm)	Maternal exposure, mice, gestation	Placental transfer, fetal growth restriction, neurobehavioral abnormalities	Placental histology, neurobehavioral assays	[141]
Gut Microbiome	Polystyrene MPs (5 μm)	Oral gavage, mice, 6 weeks	Dysbiosis (↓ Firmicutes, ↑ Bacteroidetes), increased gut permeability	16S rRNA sequencing, histology	[151]
Hematopoietic	Polystyrene NPs (50 nm)	In vitro human hematopoietic stem cells	DNA hypomethylation, impaired differentiation	Epigenetic assays, flow cytometry	[152]

Notes: ROS = reactive oxygen species.

## Data Availability

No new data were created in this review.

## References

[1] Branscomb E., Russell M.J. (2018). Frankenstein or a Submarine Alkaline Vent: Who is Responsible for Abiogenesis?. BioEssays.

[2] Brack A. (1999). Life in the solar system. Adv. Space Res..

[3] Russell M.J., Simon Duval S. (2025). Fougerite: Free Energy Converter for Life’s Conception. “Metal Ions and the Route to Life” Wolfgang Nitschke.

[4] Russell M. (2023). A Self-sustaining Serpentinization Mega-engine Feeds the Fougerite Nanoengines Implicated in the Emergence of Guided Metabolism. Front. Microbiol..

[5] Wang Y., Du Y. (2025). Hypothesis for Molecular Evolution in the Pre-Cellular Stage of the Origin of Life. Wiley Interdiscip. Rev. RNA.

[6] Russell M.J., Barge L.M., Bhartia R., Bocanegra D., Bracher P.J., Branscomb E., Kidd R., McGlynn S., Meier D.H., Nitschke W. (2014). The drive to life on wet and icy worlds. Astrobiology.

[7] Ueda H., Shibuya T. (2021). Composition of the Primordial Ocean Just after Its Formation: Constraints from the Reactions between the Primitive Crust and a Strongly Acidic, CO_2_-Rich Fluid at Elevated Temperatures and Pressures. Minerals.

[8] Smith V.J., Deshmukh M., Wallach T. (2024). The Molecular Ecology of Amniotic Fluid. J. Reprod. Immunol..

[9] Jauniaux E., Jurkovic D., Gulbis B., Collins W.P., Zaidi J., Campbell S. (1994). Investigation of the acid–base balance of coelomic and amniotic fluids in early human pregnancy. Am. J. Obstet. Gynecol..

[10] Underwood M.A., Gilbert W.M., Sherman M.P. (2005). Amniotic fluid: Not just fetal urine anymore. J. Perinatol..

[11] Suliburska J., Kocyłowski R., Komorowicz I., Grzesiak M., Bogdański P., Barałkiewicz D. (2016). Concentrations of Mineral in Amniotic Fluid and Their Relations to Selected Maternal and Fetal Parameters. Biol. Trace Elem. Res..

[12] Cooper S.J. (2008). From Claude Bernard to Walter Cannon. Emergence of the concept of homeostasis. Appetite.

[13] Billman G.E. (2020). Homeostasis: The Underappreciated and Far Too Often Ignored Central Organizing Principle of Physiology. Front. Physiol..

[14] Rasmussen J.M., Thompson P.M., Entringer S., Buss C., Wadhwa P.D. (2022). Fetal programming of human energy homeostasis brain networks: Issues and considerations. Obes. Rev..

[15] Reynolds R.M., Labad J., Buss C., Ghaemmaghami P., Raikkonen K. (2013). Transmitting biological effects of stress in utero: Implications for mother and offspring. Psychoneuroendocrinology.

[16] Martin W., Baross J., Kelley D., Russell M.J. (2008). Hydrothermal vents and the origin of life. Nat. Rev. Microbiol..

[17] Salmaso N., Tomasi S., Vaccarino F.M. (2014). Neurodevelopmental origins of health and disease. Curr. Opin. Neurol..

[18] Hammerling U. (2019). Membrane potential, the Na⁺/K⁺-ATPase and ATP consumption. Physiol. Rev..

[19] Alberts B. (2015). Molecular Biology of the Cell.

[20] Tobias A., Ballard B.D., Mohiuddin S.S. (2025). Physiology, Water Balance. [Updated 2022 Oct 3]. StatPearls.

[21] Guyton A.C., Hall J.E. (2016). Body Fluid Compartments in Adults. Textbook of Medical Physiology.

[22] Tsigos C., Chrousos G.P. (2002). Hypothalamic–pituitary–adrenal axis, neuroendocrine factors and stress. J. Psychosom. Res..

[23] Weir M.R. (2002). The renin–angiotensin–aldosterone system. Am. J. Hypertens.

[24] Verkman A.S. (2011). Roles of aquaporin-mediated water transport in fluid homeostasis. J. Physiol..

[25] Crowley S.D., Gurley S.B., Oliverio M.I., Pazmino A.K., Griffiths R., Flannery P.J., Spurney R.F., Kim H.S., Smithies O., Le T.H. (2005). Distinct roles for the kidney and systemic tissues in blood pressure regulation by the renin-angiotensin system. J. Clin. Investig..

[26] Damkier H.H., Brown P.D., Praetorius J. (2010). Epithelial pathways in choroid plexus fluid secretion. News Physiol. Sci..

[27] Ducas R., Saini B.S., Yamamura K., Bhagra C., Marini D., Silversides C.K., Roche S.L., Colman J.M., Kingdom J.C., Sermer M. (2021). Maternal and Fetal Hemodynamic Adaptations to Pregnancy and Clinical Outcomes in Maternal Cardiac Disease. Can. J. Cardiol..

[28] De Bold A.J., Borenstein H.B., Veress A.T., Sonnenberg H. (1981). A rapid and potent natriuretic response to intravenous injection of atrial myocardial extract in rats. Life Sci..

[29] Millero F.J., Feistel R., Wright D.W., McDougall T.J. (2008). The composition of Standard Seawater and the definition of the Reference-Composition Salinity Scale. Deep-Sea Res. I.

[30] Viero C., Pitard B., Rent G. (2006). Electrolyte composition and volume changes of amniotic fluid throughout gestation. Prenat. Diagn..

[31] Brace R.A. (1997). Volume, composition, and acidity of fluid recovered from fetal lungs in utero. Am. J. Obstet. Gynecol..

[32] Sojo V., Herschy B., Whicher A., Camprubí E., Lane N. (2016). The Origin of Life in Alkaline Hydrothermal Vents. Astrobiology.

[33] Waypa G.B., Marks J.D. (2012). Osmotic disruption of cell volume and membrane potential. Clin. Exp. Pharmacol. Physiol..

[34] Hohmann S., Nielsen S. (2000). Molecular Biology and Physiology of Water and Solute Transport.

[35] Sterling P. (2012). Allostasis: A model of predictive regulation. Physiol. Behav..

[36] Liu F., Gao B., Lei L., Liu S., Li H., Guo M. (2025). Intercellular flow dominates the poroelasticity of multicellular tissues. Nat. Phys..

[37] Fitzsimmons E.D., Bajaj T. (2023). Embryology, Amniotic Fluid. StatPearls.

[38] Pullano J.G., Cohen-Addad N., Apuzzio J.J., Ganesh V.L., Josimovich J.B. (1989). Water and salt conservation in the human fetus and newborn. I. Evidence for a role of fetal prolactin. J. Clin. Endocrinol. Metab..

[39] NOAA Sea Water. https://www.noaa.gov/jetstream/ocean/sea-water.

[40] LibreTexts Chemistry and Geochemistry of the Oceans. https://chem.libretexts.org/Bookshelves/Environmental_Chemistry/Geochemistry_(Lower)/02%3A_The_Hydrosphere/2.03%3A_Chemistry_and_geochemistry_of_the_oceans.

[41] EBSCOhost Seawater Composition. https://www.ebsco.com/research-starters/oceanography/seawater-composition.

[42] Tsukimori K., Fukushima K., Tsukimori M., Nakano H. (2007). Determination of reference values for electrolytes in amniotic fluid during normal pregnancy. J. Ultrasound Med..

[43] Gizzo S., Noventa M., Fagherazzi S., Lamparelli L., Ancona E., Di Gangi S., Nardelli G.B. (2012). Update on amniotic fluid: From physiological role to clinical utility. J. Matern. Fetal Neonatal Med..

[44] Brace R.A. (1983). Physiology of amniotic fluid volume regulation. Am. J. Physiol..

[45] Cole D.E., Baldwin L.S., Stirk L.J. (1992). Concentrations of sulfate in human amniotic fluid. Clin. Chim. Acta.

[46] Pressman E.K., Cavanaugh J.L., Mingione M.J., Woods J.R. (1996). Amniotic fluid acid–base balance in normal pregnancy. Am. J. Obstet. Gynecol..

[47] Pilson M.E.Q. (2013). An Introduction to the Chemistry of the Sea.

[48] Millero F.J. (2013). Chemical Oceanography.

[49] Santolaya J., Faro R. (1994). Amniotic fluid physiology. Clin. Obstet. Gynecol..

[50] Painter S.C., Sanders R., Waldron H.N., Lucas M.I., Torres-Valdes S. (2008). Urea distribution and uptake in the Atlantic Ocean between 50° N and 50° S. Mar. Ecol. Prog. Ser..

[51] Khadjeh G.H., Ranjbar R., Salehi M., Banankhojasteh S.M. (2007). Biochemical evaluation of amniotic fluid during different stages of gestation in the goat. Iranian J. Vet. Res. Univ. Shiraz.

[52] Tong X.L., Wang L., Gao T.B., Qin Y.G., Qi Y.Q., Xu Y.P. (2009). Potential function of amniotic fluid in fetal development, novel insights by comparing the composition of human amniotic fluid with umbilical cord and maternal serum at mid and late gestation. J. Chin. Med. Assoc..

[53] Chen X., Zhang Z., Xie F. (2025). Spatiotemporal dynamics of dissolved organic matter in Asia’s longest river: Linking isotopes, land use, and anthropogenic impacts. Environ. Res..

[54] Romero R., Jimenez C., Lohda A.K., Nores J., Hanaoka S., Avila C., Callahan R., Mazor M., Hobbins J.C., Diamond M.P. (1990). Amniotic fluid glucose concentration: A rapid and simple method for the detection of intraamniotic infection in preterm labor. Am. J. Obstet. Gynecol..

[55] Gluck L., Kulovich M.V. (1971). Lecithin/sphingomyelin ratios in amniotic fluid in prediction of fetal lung maturity. Am. J. Obstet. Gynecol..

[56] De Vargas C., Audic S., Henry N., Decelle J., Mahé F., Logares R., Lara E., Berney C., Le Bescot N., Probert I. (2015). Eukaryotic plankton diversity in the sunlit ocean. Science.

[57] John K. (1986). Volkman, A review of sterol markers for marine and terrigenous organic matter. Org. Geochem..

[58] Holzgreve W., Hahn S. (2002). Fetal cells in maternal circulation and cell-free fetal DNA. Hum. Reprod. Update.

[59] Koh W., Pan W., Gawad C., Fan H.C., Kerchner G.A., Wyss-Coray T., Blumenfeld Y.J., El-Sayed Y.Y., Quake S.R. (2014). Noninvasive in vivo monitoring of tissue-specific global gene expression in humans. Proc. Natl. Acad. Sci. USA.

[60] Thomsen P.F., Willerslev E. (2015). Environmental DNA—An emerging tool in conservation. Biol. Conserv..

[61] Benner R., Hansell D.A., Carlson C.A. (2014). Chemical composition and reactivity. Biogeochemistry of Marine Dissolved Organic Matter.

[62] Cho C.-K.J., Shan S.J., Winsor E.J., Diamandis E.P. (2007). Diamandis, Proteomics Analysis of Human Amniotic Fluid *. Mol. Cell. Proteom..

[63] Whitman W.B., Coleman D.C., Wiebe W.J. (1998). Prokaryotes: The unseen majority. Proc. Natl. Acad. Sci. USA.

[64] Suttle C.A. (2007). Marine viruses--major players in the global ecosystem. Nat. Rev. Microbiol..

[65] Amend A. (2014). From dandruff to deep-sea vents: Malassezia-like fungi are ecologically hyper-diverse. PLoS Pathog..

[66] Aagaard K., Ma J., Antony K.M., Ganu R., Petrosino J., Versalovic J. (2014). The placenta harbors a unique microbiome. Sci. Transl. Med..

[67] Perez-Muñoz M.E., Arrieta M.-C., Ramer-Tait A.E., Walter J. (2017). A critical assessment of the “sterile womb” and “in utero colonization” hypotheses: Implications for research on the pioneer infant microbiome. Microbiome.

[68] Kacerovsky M., Pliskova L., Menon R., Kutova R., Musilova I., Maly J., Andrys C. (2014). Microbial load of umbilical cord blood Ureaplasma species and Mycoplasma hominis in preterm prelabor rupture of membranes. J. Matern. Neonatal Med..

[69] Mor G., Kwon J.Y. (2015). Trophoblast-microbiome interaction: A new paradigm on immune regulation. Am. J. Obstet. Gynecol..

[70] Pietramellara G., Ascher J., Borgogni F., Ceccherini M.T., Guerri G., Nannipieri P. (2009). Extracellular DNA in soil and sediment: Fate and ecological relevance. Biol. Fertil. Soils..

[71] Dell’Anno A., Danovaro R. (2005). Extracellular DNA plays a key role in deep-sea ecosystem functioning. Science.

[72] Torti A., Lever M.A., Jørgensen B.B. (2015). Origin, dynamics, and implications of extracellular DNA pools in marine sediments. Mar Genom..

[73] Wang S., Tian R., Bi Y., Meng F., Zhang R., Wang C., Wang D., Liu L., Zhang B. (2024). A review of distribution and functions of extracellular DNA in the environment and wastewater treatment systems. Chemosphere.

[74] Paul J.H., Jeffrey W.H., DeFlaun M.F. (1987). Dynamics of extracellular DNA in the marine environment. Appl. Environ. Microbiol..

[75] Bianchi D.W., Chiu R.W. (2018). Sequencing of Circulating Cell-free DNA during Pregnancy. N. Engl. J. Med..

[76] Lo Y.M.D., Corbetta N., Chamberlain P.F., Rai V., Sargent I.L., Redman C.W., Wainscoat J.S. (1997). Presence of fetal DNA in maternal plasma and serum. Lancet.

[77] Finning K., Martin P., Summers J., Massey E., Poole G., Daniels G. (2002). Fetal genotyping for the KEL1 and RHD alleles by analysis of maternal plasma DNA. Transfusion.

[78] Tsui N.B.Y., Chiu R.W.K., Lo Y.M.D. (2002). Epigenetic approaches for the analysis of fetal nucleic acids in maternal plasma. Trends Mol. Med..

[79] Halfar J., Čabanová K., Vávra K., Delongová P., Motyka O., Špaček R., Kukutschová J., Šimetka O., Heviánková S. (2023). Microplastics and additives in patients with preterm birth: The first evidence of their presence in both human amniotic fluid and placenta. Chemosphere.

[80] Tian J., Liang L., Li Q., Li N., Zhu X., Zhang L. (2025). Association between microplastics in human amniotic fluid and pregnancy outcomes: Detection and characterization using Raman spectroscopy and pyrolysis GC/MS. J. Hazard. Mater..

[81] Pabortsava E., Lampitt R.S. (2020). High concentrations of plastic hidden beneath the surface of the Atlantic Ocean. Nat. Commun..

[82] Eriksen M., Lebreton L.C., Carson H.S., Thiel M., Moore C.J., Borerro J.C., Galgani F., Ryan P.G., Reisser J. (2014). Plastic Pollution in the World’s Oceans: More than 5 Trillion Plastic Pieces Weighing over 250,000 Tons Afloat at Sea. PLoS ONE.

[83] Cózar A., Echevarría F., González-Gordillo J.I., Irigoien X., Ubeda B., Hernández-León S., Palma A.T., Navarro S., García-de-Lomas J., Ruiz A. (2014). Plastic debris in the open ocean. Proc. Natl. Acad. Sci. USA.

[84] van Sebille E., Wilcox C., Lebreton L., Maximenko N., Hardesty B.D., A van Franeker J., Eriksen M., Siegel D., Galgani F., Law K.L. (2015). A global inventory of small floating plastic debris. Environ. Res. Lett..

[85] Banchi P., Colitti B., Opsomer G., Rota A., Van Soom A. (2023). The dogma of the sterile uterus revisited: Does microbial seeding occur during fetal life in humans and animals?. Reproduction.

[86] Banchi P., Bertero A., Corrò M., Colitti B., Maniscalco L., Van Soom A., Rota A. (2025). Approaching the sterile womb theory in dogs and cats: A multi-technique investigation. Theriogenology.

[87] Panzer J.J., Romero R., Greenberg J.M., Winters A.D., Galaz J., Gomez-Lopez N., Theis K.R. (2023). Is there a placental microbiota? A critical review and re-analysis of published placental microbiota datasets. BMC Microbiol..

[88] Płotka-Wasylka J., Mulkiewicz E., Lis H., Godlewska K., Kurowska-Susdorf A., Sajid M., Lambropoulou D., Jatkowska N. (2023). Endocrine disrupting compounds in the baby’s world—A harmful environment to the health of babies. Sci. Total Environ..

[89] Yi J., Ma Y., Ruan J., You S., Ma J., Yu H., Zhao J., Zhang K., Yang Q., Jin L. (2024). The invisible Threat: Assessing the reproductive and transgenerational impacts of micro- and nanoplastics on fish. Environ. Int..

[90] Dai Y., Han R., Yao Z., Yan H., Liu Z., Liu X., Yue T., Zhao J., Wang Z., Xing B. (2025). Intergenerational transfer of micro(nano)plastics in different organisms. J. Hazard. Mater..

[91] Jambeck J.R., Geyer R., Wilcox C., Siegler T.R., Perryman M., Andrady A., Narayan R., Law K.L. (2015). Plastic waste inputs from land into the ocean. Science.

[92] Van Cauwenberghe L., Janssen C.R. (2014). Microplastics in bivalves cultured for human consumption. Environ. Pollut..

[93] Li J., Yang D., Li L., Jabeen K., Shi H. (2015). Microplastics in commercial bivalves from China. Environ. Pollut..

[94] Yang D., Shi H., Li L., Li J., Jabeen K., Kolandhasamy P. (2015). Microplastic Pollution in Table Salts from China. Environ. Sci. Technol..

[95] Qian N., Gao X., Lang X., Deng H., Bratu T.M., Chen Q., Stapleton P., Yan B., Min W. (2024). Rapid single-particle chemical imaging of nanoplastics by SRS microscopy. Proc. Natl. Acad. Sci. USA.

[96] Inaudi P., Sicurella G.M., Rivoira L., Favilli L., Bracco P., Bertinetti S., Abollino O., Bruzzoniti M.C., Isaja V., Giacomino A. (2025). Pollution profiling in Italian honeys: Elements and microplastics as comprehensive indicators of environmental contamination and food safety. Sci. Total Environ..

[97] Nabawy N.M., Ibrahim S.A., Abd El-Hameid N.A., Ghonemy O.I., Shaalan W.M. (2025). Effects of Microplastics on Gene Expression, Muscular Performance, and Immunological Responses in Nile Tilapia (*Oreochromis niloticus*): Seasonal and Habitat Variations. Mar. Biotechnol..

[98] Wang Z., Fan L., Wang J., Xie S., Zhang C., Zhou J., Zhang L., Xu G., Zou J. (2021). Insight into the immune and microbial response of the white-leg shrimp Litopenaeus vannamei to microplastics. Mar. Environ. Res..

[99] Liebezeit G., Liebezeit E. (2014). Synthetic particles as contaminants in German beers. Food Addit. Contam. Part A.

[100] Yue Z., Liu X., Mei T., Zhang Y., Pi F., Dai H., Zhou Y., Wang J. (2024). Reducing microplastics in tea infusions released from filter bags by pre-washing method: Quantitative evidences based on Raman imaging and Py-GC/MS. Food Chem..

[101] Prata J.C., Paço A., Reis V., da Costa J.P., Fernandes A.J.S., da Costa F.M., Duarte A.C., Rocha-Santos T. (2020). Identification of microplastics in white wines capped with polyethylene stoppers using micro-Raman spectroscopy. Food Chem..

[102] Makhdoumi P., Pirsaheb M., Amin A.A., Kianpour S., Hossini H. (2025). Microplastic pollution in table salt and sugar: Occurrence, qualification and quantification and risk assessment. J. Food Compos. Anal..

[103] Visentin E., Niero G., Benetti F., O’Donnell C., De Marchi M. (2025). Assessing microplastic contamination in milk and dairy products. NPJ Sci. Food.

[104] Wang H.P., Huang X.H., Chen J.N., Dong M., Zhang Y.Y., Qin L. (2023). Pouring hot water through drip bags releases thousands of microplastics into coffee. Food Chem..

[105] Nguyen V.T.T., Hoang H.M., Duong D.D. (2025). Micro-and nanoplastic contamination in beverages in Vietnam. Environ. Monit. Assess..

[106] Dris R., Gasperi J., Mirande C., Mandin C., Guerrouache M., Langlois V., Tassin B. (2017). A first overview of textile fibers, including microplastics, in indoor and outdoor environments. Environ. Pollut..

[107] Dewika M., Markandan K., Nagaratnam S., Irfan N.A., Abdah M.A.A.M., Ruwaida J.N., Sara Y.Y., Khalid M. (2025). Assessing the concentration, distribution and characteristics of suspended microplastics in the Malaysian indoor environment. Sci. Total Environ..

[108] Zheng H., Guo H., Fu H., Yao K. (2024). Microplastics in indoor and outdoor environments in China: Characteristic and human exposure risk assessment. Ecotoxicol. Environ. Saf..

[109] Torres-Agullo A., Karanasiou A., Moreno T., Lacorte S. (2022). Airborne microplastic particle concentrations and characterization in indoor urban microenvironments. Environ. Pollut..

[110] Coşgun M.S., Gündoğdu S., Şahin Ü.A., Ayvaz B.U., Onat B., Ayvaz C. (2025). Microplastics in the indoor air of subway station in Istanbul. Air Qual. Atmos. Health.

[111] Akhbarizadeh R., Dobaradaran S., Amouei Torkmahalleh M., Saeedi R., Aibaghi R., Faraji Ghasemi F. (2021). Suspended fine particulate matter (PM_2.5_), microplastics (MPs), and polycyclic aromatic hydrocarbons (PAHs) in air: Their possible relationships and health implications. Environ. Res..

[112] Soltani N.S., Taylor M.P., Wilson S.P. (2021). Quantification and exposure assessment of microplastics in Australian indoor house dust. Environ. Pollut..

[113] Brown E., MacDonald A., Allen S., Allen D. (2023). The potential for a plastic recycling facility to release microplastic pollution and possible filtration remediation effectiveness. J. Hazard. Mater. Adv..

[114] Mokammel A., Naddafi K., Has-sanvand M.S., Nabizadeh R., Faridi S., Noruzzade E., Yaghmaeian K. (2025). Airborne microplastics pollution in municipal solid waste processing and disposal complex: Concentration, characterization, and composition. Emerg. Contam..

[115] Novotna J., Tunak M., Militky J., Kremenakova D., Wiener J., Novakova J., Sevcu A. (2025). Release of Microplastic Fibers from Polyester Knit Fleece during Abrasion, Washing, and Drying. ACS Omega.

[116] Yang Z., Lü F., Zhang H., Wang W., Shao L., Ye J., He P. (2021). Is incineration the terminator of plastics and microplastics?. J. Hazard. Mater..

[117] Mutshekwa T., Mulaudzi F., Maiyana V.P., Mofu L., Munyai L.F., Murungweni F.M. (2025). Atmospheric deposition of microplastics in urban, rural, forest environments: A case study of Thulamela Local Municipality. PLoS ONE.

[118] Zhang J., Wang L., Kannan K. (2020). Microplastics in house dust from 12 countries and associated human exposure. Environ. Int..

[119] Not C., Chan K., So M.W.K., Lau W., Tang L.T., Cheung C.K.H. (2025). State of microbeads in facial scrubs: Persistence and the need for broader regulation. Environ. Sci. Pollut. Res. Int..

[120] Bikiaris N., Nikolaidis N.F., Barmpalexis P. (2024). Microplastics (MPs) in Cosmetics: A Review on Their Presence in Personal-Care, Cosmetic, and Cleaning Products (PCCPs) and Sustainable Alternatives from Biobased and Biodegradable Polymers. Cosmetics.

[121] Esmeralda V.G., Patterson J., Shelciya S. (2025). Preliminary study on the ejection of microplastics from different types of face masks. J. Occup. Environ. Hyg..

[122] Gao Z., Wontor K., Cizdziel J.V., Lu H. (2022). Distribution and characteristics of microplastics in beach sand near the outlet of a major reservoir in north Mississippi, USA. Micropl. Nanopl..

[123] Kwon S., Zambrano M.C., Venditti R.A., Frazier R., Zambrano F., Gonzalez R.W., Pawlak J.J. (2022). Microfiber shedding from nonwoven materials including wipes and meltblown nonwovens in air and water environments. Environ. Sci. Pollut. Res..

[124] Napper I.E., Thompson R.C. (2016). Release of synthetic microplastic plastic fibres from domestic washing machines: Effects of fabric type and washing conditions. Mar. Pollut. Bull..

[125] Masciarelli E., Casorri L., Di Luigi M., Beni C., Valentini M., Costantini E., Aielli L., Reale M. (2025). Microplastics in Agricultural Crops and Their Possible Impact on Farmers’ Health: A Review. Int. J. Environ. Res. Public Health.

[126] Savva K., Llorca M., Borrell X., Bertran-Solà O., Farré M., Moreno T. (2024). Granulated rubber in playgrounds and sports fields: A potential source of atmospheric plastic-related contaminants and plastic additives after runoff events. J. Hazard. Mater..

[127] Fang C., Zhou W., Hu J., Wu C., Niu J., Naidu R. (2024). Paint has the potential to release microplastics, nanoplastics, inorganic nanoparticles, and hybrid materials. Environ. Sci. Eur..

[128] Lau W.W.Y., Shiran Y., Bailey R.M., Cook E., Stuchtey M.R., Koskella J., Velis C.A., Godfrey L., Boucher J., Murphy M.B. (2020). Evaluating scenarios toward zero plastic pollution. Science.

[129] Landrigan P.J., Raps H., Cropper M., Bald C., Brunner M., Canonizado E.M., Charles D., Chiles T.C., Donohue M.J., Enck J. (2023). The Minderoo-Monaco Commission on Plastics and Human Healt. Ann. Glob. Health.

[130] Chauhan R., Archibong A.E., Ramesh A. (2023). Imprinting and Reproductive Health: A Toxicological Perspective. Int. J. Mol. Sci..

[131] Rubin A.E., Zucker I. (2022). Interactions of microplastics and organic compounds in aquatic environments: A case study of augmented joint toxicity. Chemosphere.

[132] Zhang Y.J., Guo J.L., Xue J.C., Bai C.L., Guo Y. (2021). Phthalate metabolites: Characterization, toxicities, global distribution, and exposure assessment. Environ. Pollut..

[133] Ragusa A., Svelato A., Santacroce C., Catalano P., Notarstefano V., Carnevali O., Papa F., Rongioletti M.C.A., Baiocco F., Draghi S. (2021). Plasticenta: First evidence of microplastics in human placenta. Environ. Int..

[134] Ragusa A., Notarstefano V., Svelato A., Belloni A., Gioacchini G., Blondeel C., Zucchelli E., De Luca C., D’avino S., Gulotta A. (2022). Raman Microspectroscopy Detection and Characterisation of Microplastics in Human Breastmilk. Polymers.

[135] Ragusa A., Cristiano L., Di Vinci P., Familiari G., Nottola S.A., Macchiarelli G., Svelato A., De Luca C., Rinaldo D., Neri I. (2025). Artificial plasticenta: How polystyrene nanoplastics affect in-vitro cultured human trophoblast cells. Front. Cell Dev. Biol..

[136] Lopez G.L., Lamarre A. (2025). The impact of micro- and nanoplastics on immune system development and functions: Current knowledge and future directions. Reprod. Toxicol..

[137] Liu S., Liu X., Guo J., Yang R., Wang H., Sun Y., Chen B., Dong R. (2023). The Association Between Microplastics and Microbiota in Placentas and Meconium: The First Evidence in Humans. Environ. Sci. Technol..

[138] Amereh F., Amjadi N., Mohseni-Bandpei A., Isazadeh S., Mehrabi Y., Eslami A., Naeiji Z., Rafiee M. (2022). Placental plastics in young women from general population correlate with reduced foetal growth in IUGR pregnancies. Environ. Pollut..

[139] Prüst M., Meijer J., Westerink R.H.S. (2020). The plastic brain: Neurotoxicity of micro- and nanoplastics. Part. Fibre Toxicol..

[140] So Y.H., Shin H.S., Lee S.H., Moon H.J., Jang H.J., Lee E.-H., Jung E.-M. (2023). Maternal exposure to polystyrene microplastics impairs social behavior in mouse offspring with a potential neurotoxicity. NeuroToxicology.

[141] Chen J., Yan L., Zhang Y., Liu X., Wei Y., Zhao Y., Li K., Shi Y., Liu H., Lai W. (2024). Maternal exposure to nanopolystyrene induces neurotoxicity in offspring through P53-mediated ferritinophagy and ferroptosis in the rat hippocampus. J. Nanobiotechnol..

[142] Ma Q., Lei J., Pang Y., Shen Y., Zhang T. (2025). Nanoplastics: AComprehensive Review of Central Nervous System Impacts. Env. Health.

[143] Wu X., Leung T., Jima D.D., Iyangbe M., Bang J. (2025). Developing a feasible fast-track testing method for developmental neurotoxicity studies: Alternative model for risk assessment of micro- and nanoplastics. Front. Toxicol..

[144] Sharma A., Kaur M., Sharma K., Bunkar S.K., John P., Bhatnagar P. (2023). Nano polystyrene induced changes in anxiety and learning behaviour are mediated through oxidative stress and gene disturbance in mouse brain regions. Neurotoxicology.

[145] Wang Q., Wu Y., Zhang W., Shen T., Li H., Wu J., Zhang L., Qin L., Chen R., Gu W. (2022). Lipidomics and transcriptomics insight into impacts of microplastics exposure on hepatic lipid metabolism in mice. Chemosphere.

[146] Wang Y.L., Lee Y.H., Hsu Y.H., Chiu I.J., Huang C.C., Huang C.C., Chia Z.C., Lee C.P., Lin Y.F., Chiu H.W. (2021). The Kidney-Related Effects of Polystyrene Microplastics on Human Kidney Proximal Tubular Epithelial Cells HK-2 and Male C57BL/6 Mice. Environ. Health Perspect..

[147] Hirt N., Body-Malapel M. (2020). Immunotoxicity and intestinal effects of nano- and microplastics: A review of the literature. Part. Fibre Toxicol..

[148] Wei W., Li Y., Lee M., Andrikopoulos N., Lin S., Chen C., Leong D.T., Ding F., Song Y., Ke P.C. (2022). Anionic nanoplastic exposure induces endothelial leakiness. Nat. Commun..

[149] Feng Y., Yuan H., Wang W., Xu Y., Zhang J., Xu H., Fu F. (2022). Co-exposure to polystyrene microplastics and lead aggravated ovarian toxicity in female mice via the PERK/eIF2α signaling pathway. Ecotoxicol. Environ. Saf..

[150] Liu Y., Hao F., Liang H., Liu W., Guo Y. (2025). Exposure to polystyrene nanoplastics impairs sperm metabolism and pre-implantation embryo development in mice. Front. Cell Dev. Biol..

[151] Lu L., Wan Z., Luo T., Fu Z., Jin Y. (2018). Polystyrene microplastics induce gut microbiota dysbiosis and hepatic lipid metabolism disorder in mice. Sci. Total Environ..

[152] Malinowska K., Tarhonska K., Foksiński M., Sicińska P., Jabłońska E., Reszka E., Zarakowska E., Gackowski D., Górecka K., Balcerczyk A. (2024). Impact of Short-Term Exposure to Non-Functionalized Polystyrene Nanoparticles on DNA Methylation and Gene Expression in Human Peripheral Blood Mononuclear Cells. Int. J. Mol. Sci..

[153] Ullah F., Wang P.Y., Saqib S., Zhao L., Ashraf M., Khan A., Khan W., Khan A., Chen Y., Xiong Y.C. (2025). Toxicological complexity of microplastics in terrestrial ecosystems. iScience.

[154] Habumugisha T., Zhang Z., Uwizewe C., Yan C., Ndayishimiye J.C., Rehman A., Zhang X. (2024). Toxicological review of micro- and nano-plastics in aquatic environments: Risks to ecosystems, food web dynamics and human health. Ecotoxicol. Environ. Saf..

[155] WHO One Health. https://www.who.int/health-topics/one-health#tab=tab_1.

[156] Pitt S.J., Gunn A. (2024). The One Health Concept. Br. J. Biomed. Sci..

[157] MacKendrick N. (2014). Plastic childhood: An environmental sociology of toys. Sociol. Perspect..

[158] Sunyach C., Antonelli B., Tardieu S., Marcot M., Perrin J., Bretelle F. (2018). Environmental Health in Perinatal and Early Childhood: Awareness, Representation, Knowledge and Practice of Southern France Perinatal Health Professionals. Int. J. Environ. Res. Public Health.

[159] Trasande L., Massey R.I., DiGangi J., Geiser K., Olanipekun A.I., Gallagher L. (2011). How Developing Nations Can Protect Children From Hazardous Chemical Exposures While Sustaining Economic Growth. Health Aff..

[160] Rummel C.D., Löder M.G., Fricke N.F., Lang T., Griebeler E.M., Janke M., Gerdts G. (2016). Plastic ingestion by pelagic and demersal fish from the North Sea and Baltic Sea. Mar. Pollut. Bull..

[161] Rockström J., Steffen W., Noone K., Persson A., Chapin F.S., Lambin E.F., Lenton T.M., Scheffer M., Folke C., Schellnhuber H.J. (2009). A safe operating space for humanity. Nature.

[162] 171 Rist S., Almroth B.C., Hartmann N.B., Karlsson T.M. (2018). A critical perspective on early communications concerning human health aspects of microplastics. Sci. Total Environ..

[163] Sharma S., Chatterjee S. (2017). Microplastic pollution, a threat to marine ecosystem and human health: A short review. Environ. Sci. Pollut. Res..

[164] Lusher A.L., Hollman P., Mendoza-Hill J.J. (2017). Microplastics in Fisheries and Aquaculture: Status of Knowledge on Their Occurrence and Implications for Aquatic Organisms and Food Safety.

[165] Santillo D., Miller K., Johnston P. (2022). Microplastic contamination in aquatic environments: Source, fate and potential impacts on marine organisms. Mar. Pollut. Bull..

[166] Zinsstag J., Schelling E., Waltner-Toews D., Tanner M. (2011). From “one medicine” to “one health” and systemic approaches to health and well-being. Prev. Vet. Med..

[167] Destoumieux-Garzón D., Mavingui P., Boetsch G., Boissier J., Darriet F., Duboz P., Fritsch C., Giraudoux P., Le Roux F., Morand S. (2018). The One Health Concept: 10 Years Old and a Long Road Ahead. Front. Vet. Sci..

[168] Rock M., Buntain B.J., Hatfield J., Hallgrimsson B. (2019). The Evolution of One Health: A Decade of Progress and Challenges for the Future. Front. Vet. Sci..

[169] Cox K.D., Covernton G.A., Davies H.L., Dower J.F., Juanes F., Dudas S.E. (2019). Human Consumption of Microplastics. Environ Sci. Technol..

[170] Landrigan P.J. (2018). Pollution and children’s health. Sci. Total Environ..

[171] Cordiner M.A., Gibb E.L., Kisiel Z., Roth N.X., Biver N., Bockelée-Morvan D., Boissier J., Bonev B.P., Charnley S.B., Coulson I.M. (2025). A D/H ratio consistent with Earth’s water in Halley-type comet 12P from ALMA HDO mapping. Nat. Astron..

[172] Ragusa A., Principi G., Matta M. (2022). Pregnancy in the Era of the Environmental Crisis: Plastic and Pollution. Clin. Exp. Obstet. Gynecol..

[173] Ragusa A., De Luca C., Zucchelli E., Rinaldo D., Svelato A. (2023). Plastic, microplastic, and the inconsistency of human thought. Front. Public Health.

[174] Serrano-Aguirre L., Prieto M.A. (2024). Can bioplastics always offer a truly sustainable alternative to fossil-based plastics?. Microb. Biotechnol..

[175] McVeigh K., Bryce E. Plastic Pollution Talks Fail as Negotiators in Geneva Reject Draft Treaties. First Published on Friday 15 August 2025. https://www.theguardian.com/environment/2025/aug/15/plastic-pollution-talks-geneva-treaty.

[176] United Nations Development Programme (2024). Combatting Plastic Pollution for Sustainable Development: A Snapshot of UNDP’s Work in 12 Countries.

